# Innovations in anaerobic digestion: a model-based study

**DOI:** 10.1186/s13068-020-01864-z

**Published:** 2021-01-13

**Authors:** Karol Postawa, Jerzy Szczygieł, Marek Kułażyński

**Affiliations:** Faculty of Chemistry, Wrocław University of Science and Technology, Wybrzeż Wyspiańskiego 27, 50-370 Wrocław, Poland

**Keywords:** Biogas, Intensification, Temperature-phased anaerobic digestion, Autogenerative high-pressure digestion, Case study, Modeling

## Abstract

**Background:**

Increasing the efficiency of the biogas production process is possible by modifying the technological installations of the biogas plant. In this study, specific solutions based on a mathematical model that lead to favorable results were proposed. Three configurations were considered: classical anaerobic digestion (AD) and its two modifications, two-phase AD (TPAD) and autogenerative high-pressure digestion (AHPD). The model has been validated based on measurements from a biogas plant located in Poland. Afterward, the TPAD and AHPD concepts were numerically tested for the same volume and feeding conditions.

**Results:**

The TPAD system increased the overall biogas production from 9.06 to 9.59%, depending on the feedstock composition, while the content of methane was slightly lower in the whole production chain. On the other hand, the AHPD provided the best purity of the produced fuel, in which a methane content value of 82.13% was reached. At the same time, the overpressure leads to a decrease of around 7.5% in the volumetric production efficiency. The study indicated that the dilution of maize silage with pig manure, instead of water, can have significant benefits in the selected configurations. The content of pig slurry strengthens the impact of the selected process modifications—in the first case, by increasing the production efficiency, and in the second, by improving the methane content in the biogas.

**Conclusions:**

The proposed mathematical model of the AD process proved to be a valuable tool for the description and design of biogas plant. The analysis shows that the overall impact of the presented process modifications is mutually opposite. The feedstock composition has a moderate and unsteady impact on the production profile, in the tested modifications. The dilution with pig manure, instead of water, leads to a slightly better efficiency in the classical configuration. For the TPAD process, the trend is very similar, but the AHPD biogas plant indicates a reverse tendency. Overall, the recommendation from this article is to use the AHPD concept if the composition of the biogas is the most important. In the case in which the performance is the most important factor, it is favorable to use the TPAD configuration.

## Background

**Fig. 1:**
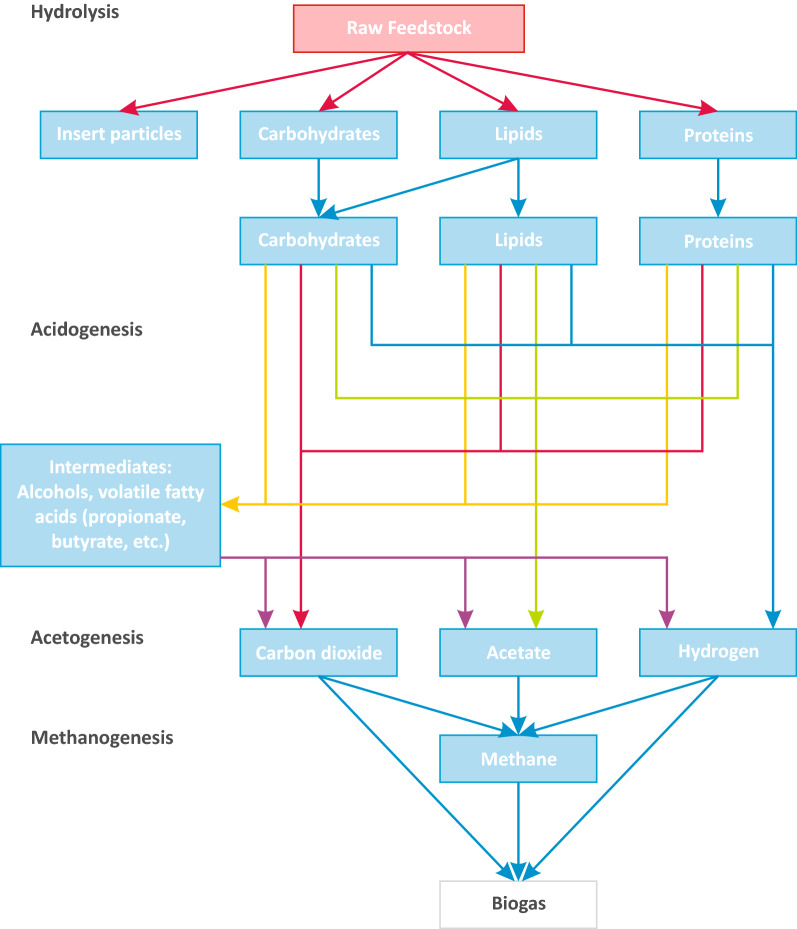
Schematic representation of the decomposition pathway in anaerobic digestion

The competition in the biofuel sector is very intense [1], which results in a directional search for new solutions to increase the production efficiency [2]. The synthesis process needs to be economically profitable and justified, not only in comparison to other bioenergy sources, but also to traditional fossil fuels. A biofuel with a very wide selection of developed and described production improvements and upgrades, is the biogas. This fuel is generated in a process called anaerobic digestion (AD)—usually described as a four-stage conversion. The simplified conversion scheme is presented in Fig. 1, however some of presented substances, can be created in more than one stage—in this case, they are listed only in the place, where the production is the highest in typical conditions.

In the first stage, the raw feedstock, consisting of mainly carbohydrates, proteins, and lipids is hydrolyzed to simpler compounds: sugars, amino acids, and long-chain fatty acids [3]. This process is driven by facultative anaerobes, mainly by extracellular enzymes [4]; thus, the process is very nonspecific [5]. This has led to a commonly used approach, which is based on separating this step from the production chain. This can be made by performing a biological pre-treatment of the raw material before injecting it to the main reactor, or by performing the physicochemical conversion in a separate unit process [6–9].

The next three steps—acidogenesis, acetogenesis, and methanogenesis—are strictly biological, and cannot be simply replaced by physical or chemical methods. However, it does not mean that they are independent of the environmental conditions, such as pressure, temperature, or pH. The acidogenesis is a process in which the acidogenic bacteria convert the products from the previous step to short-chain fatty acids, alcohols, and aromatic compounds [10]. This process, as also all of its successors, is mainly performed by intracellular enzymes [11].

During acetogenesis, the feedstock is further digested to acetate. The main substrates are fatty acids; however, in favorable conditions, the conversion of carbon dioxide can also be recycled to acetate [12, 13]. This acid is then converted by facultative anaerobes (archaeons) to methane through a process called methanogenesis [14]. However, methanol and carbon dioxide can be also a carbon source for this synthesis. As a result, $\hbox {CO}_{{2}}$ can be either a substrate or a product, depending on the pathway followed. In this case, the most important role is played by hydrogenotrophic methanogens [15]. This aspect is particularly significant in the processes under increased pressure, which will be discussed later.

As the last three steps are strictly biological and interrelated, they can’t be simply separated from the production chain, as in the case of hydrolysis. However, they can still be improved by additional modifications. These include, but are not limited to, changes in the physical properties of the bioreactor. An example of this kind of approach is using two-phase AD (TPAD). This acronym is sometimes also defined as temperature-phased AD, as the most common way to achieve the phase separation is to use two reactors in series, each one at a different temperature [16]. The environmental conditions in the first tank favor hydrolysis—the temperature is higher, usually around 65$^{\circ }$C, while the second one is kept at around 35$^{\circ }$C [17]. The lower temperature is beneficial for the last three steps. The separation has also an impact on the pH inside the reactors. In the second stage, the pH is usually neutral [18], while in the first stage, depending on the feedstock, it can be neutral (NT-TPAD) or acid (AT-TPAD) [19]. The TPAD system is significantly less popular than single-phase reactors. For example, around 95% of the AD units in Europe are declared to be single-step [20]. Recent industrial approaches in this matter, are mostly focused on pilot-scale [21, 22], while full-scale installations are still very uncommon worldwide. This study will intent to test, if a redesign of existing full-scale installation to the modified AD system, could be beneficial.

An interesting alternative to TPAD is another two-stage process, in which the differentiating factor is the pressure, instead of the temperature. This system is called autogenerative high-pressure digestion (AHPD). The production of biogas leads to a self-increase of pressure if the collection of fuel is delayed. Consequently, it is possible to increase this parameter up to 20 bar [23]. Compared to a change in temperature, a pressure change does not have such a direct impact on the biological part of the process. However, it influences the final composition of the biogas. This is because Henry’s law constants for methane and carbon dioxide don’t change proportionally with pressure—0.0016 mol/L/bar and 0.318 mol/L/bar, respectively [24, 25]. The solubility of $\hbox {CO}_{{2}}$ will rise more rapidly with pressure, than in the case of methane. This leads to a higher concentration of methane in the biogas, even reaching a 90% [24]. The high pressure is usually kept only in the first reactor in the process chain, while the second is operated at atmospheric pressure. This enables the obtention of a very pure product from first step, while achieving a good chemical oxygen demand (COD) removal in the overall system.

It would be beneficial to introduce both of the described “production-step” modifications on an industrial scale; however, they need to be preceded by initial tests. This can be done using mathematical models of the process [26]. This study aims to demonstrate the practical application of a mathematical model (of an AD process) developed by our team [27, 28], which after successful verification, can be used to optimize the operation of real systems and formulate recommendations of changes for the tested real installations. In this study, a model-based comparison will be performed to test which of the proposed modifications could potentially improve the economic efficiency of an existing two-step AD unit, with a total volume of 6600 $\hbox {m}^{3}$.

## Results and discussion

### The results of modeling for the initial configuration

The first series of model trials was intended to prove that the prepared model, numerical description of the feedstock, as well as the other model inputs, were correct. All four mentioned initial cases, for which experimental data are available, were reproduced using the model. The results were then compared with this data and presented in Table 1.

**Table 1:** Summary of model validation

	Maize silage	Pig manure	Water	Maize silage	Pig manure	Water
	Case 1			Case 2		
Feedstock [%]	51.61	0.00	48.39	52.38	0.00	47.62
	Biogas [Nm$^{{3}}$/d]	Methane [%]		Biogas [Nm$^{{3}}$/d]	Methane [%]	
Experiment	12,114.80	54.06		12,468.80	54.16	
Model	11,916.36	55.36		12,052.70	55.34	
Relative error [%]	1.64	2.41		3.34	2.18	
	Case 3			Case 4		
Feedstock [%]	50.82	49.18	0.00	51.61	48.39	0.00
	Biogas [Nm$^{{3}}$/d]	Methane [%]		Biogas [Nm$^{{3}}$/d]	Methane [%]	
Experiment	11,878.00	54.30		12,392.26	54.07	
Model	11,786.09	56.42		11,908.66	56.36	
Relative error [%]	0.77	3.90		3.90	4.25	

As can be seen from Table 1, the model reproduces the process very well. The relative error of the model for process efficiency (daily biogas volumetric flux) and biogas quality (methane content in biogas) does not exceed 3.9 and 4.25%, respectively. Besides, there are no visible trends between the input composition and the relative error value. This indicates that, in the preparation of the feedstock description, there were no directional errors.

The dilution of the maize silage with pig manure, instead of water, did not significantly benefit the classical configuration. Its comparison with the case of an equal proportion of water and manure (Case 1 and Case 4) shows that the differences in the composition of biogas are negligible (0.01 percentage point), and which are in the same order of magnitude as the measurement accuracy and fluctuations. The volume flux of produced biogas is slightly higher for manure-diluted feedstock; however, this difference is lower than 2.5% (also Case 1 and Case 4) of the total production, making it insignificant, and it would only be beneficial if pig manure was a waste material available in the close surroundings of the biogas plant.

### The results for process modifications and summary

The series of simulations for AHPD and TPAD configurations were performed. As the experimental results were not available for them, the model was utilized as a tool to provide all necessary data for the analysis. Both concepts were considered independently, in the same order as in the initial trial. To increase the resolution of the method, the production in both reactors, for every production chain, were described separately. The results are presented in Table 2.

**Table 2:** Summary of simulations for alternative concepts

Case 1 (51.61% MS, 48.39% W)			Case 2 (52.38% MS, 47.62% W)		
AHPD	${\text{Biogas}} [{Nm}^{{3}}{/d}]$	Methane [%]	AHPD	${\text{Biogas}} [{Nm}^{{3}}{/d}]$	Methane [%]
R1 (*p* = 20.27 bar)	7430.42	79.3	R1 (*p* = 20.27 bar)	7623.87	78.79
R2 (*p* = 1.013 bar)	3702.05	19.56	R2 (*p* = 1.013 bar)	3688.94	19.27
Total	11,132.47	59.43	Total	11,312.81	59.38
TPAD	${\text{Biogas}} [{Nm}^{{3}}{/d}]$	Methane [%]	TPAD	${\text{Biogas}} [{Nm}^{{3}}{/d}]$	Methane [%]
${R1 (\textit{T}=55^{\circ }C)}$	10,719.03	50.26	${R1 (\textit{T}=55^{\circ }C)}$	10,830	50.25
${R2 (\textit{T}=39^{\circ }C)}$	2279.89	53.21	${R2 (\textit{T}=39^{\circ }C)}$	2314.17	53.17
Total	12,998.92	50.78	Total	13,144.17	50.76
Case 3 (50.82% MS, 49.18% PM)			Case 4 (51.61% MS, 48.39% PM)		
AHPD	$\text{Biogas} {[Nm}^{{3}}{/d]}$	Methane [%]	AHPD	$\text{Biogas}{[Nm}^{{3}}{/d]}$	Methane [%]
R1 (*p* = 20.27 bar)	7220.36	82.13	R1 (*p* = 20.27 bar)	7378.81	81.66
R2 (*p* = 1.013 bar)	3626.5	19.57	R2 (*p* = 1.013 bar)	3626.04	19.29
Total	10,846.85	61.21	Total	11,004.86	61.11
TPAD	$\text{Biogas}{[Nm}^{{3}}{/d]}$	Methane [%]	TPAD	$\text{Biogas} {[Nm}^{{3}}{/d]}$	Methane [%]
${R1 (T=55^{\circ }C)}$	10,978.98	51.02	${R1 (T=55^{\circ }C)}$	11,074.63	50.99
${R2 (T=39^{\circ }C)}$	1936.95	54.43	${R2 (\textit{T}=39^{\circ }C)}$	1,971.15	54.35
Total	12,915.92	51.53	Total	13,045.78	51.5

Starting from the AHPD concept, the biogas production is more intense in the first tank, in all cases. This was an expected result, as the first tank is intended to take over the main production burden in this type of process. The average production in the first reactor was two times higher than in the second. Moreover, the high-pressure tanks were producing a fuel with a better composition, reaching even over 82% of methane content. For comparison, for all cases, this value did not exceed 20%, in the second tank.

The impact of the feedstock composition shows an opposite dependence than in the initial, classical configuration. There is a visible relation between the composition of the biogas, especially in the first tank in the production chain, and the substance used for dilution. By using manure-diluted feedstock, around 3 percentage points more methane could be achieved in the outlet flux (Table 2: case 1 and case 4). However, in the case of the second tank in the production chain, this impact is significantly lower. Considering the production volume flux, it should be noticed that water-diluted feedstock reactors indicate a somewhat higher biogas production in both reactors (Table 2: case 1 and case 2). Thus, the selection of raw materials, in this case, should be defined by the objective: if the most important parameter is the composition of the fuel, the dilution by using manure is recommended.

The second considered configuration was TPAD. Again, the biggest part of the production occurs in tank 1, in all cases. The difference is even more noticeable than in the AHPD system: this time, up to over 4 times more biogas is produced from the first reactor. However, regarding the biogas composition, the trend is the opposite. A higher methane content can be noticed in the second tank, while the difference is not so sharp as in the AHPD. In the first compartment, it varied between 50 to 51%, while in the second, it varied from 53 to 54.5%.

The impact of feedstock composition is very similar to the previous configuration. Again, the reactors with biomass diluted using manure (Table 2: case 3 and case 4) indicated a higher methane content than water-diluted biomass. In this test, the difference was noticeable lower, but still clearly visible in both tanks. Regarding the overall biogas production flux, there is no simple dependency between this and the selected dilution factor. In general, the higher maize silage content leads to a higher efficiency of production.

The most crucial part of this analysis is the comparison between the proposed process modifications and the initial case. The differences were indicated in the relation to the modeled results—both in the classical and the modified approaches. As the character of the tested processes is very heterogeneous, in some cases, the biogas composition between the steps does not vary significantly, whereas in other cases, the difference is significant. The mixture of both streams was assumed to be the total production of every installation and considered in this form. Following these assumptions, some insights can be noticed.

First, the overall biogas production in the AHPD was lower for all feedstock compositions, than that in the initial classical configuration without overpressure (Tables 1 and 2). The difference was even more significant for the feeds containing pig manure. The decrease in the output biogas flux was even around 8%. On the other hand, the amount of methane in this fuel was higher: up to 8.5%. Generally, the cases with the lowest production indicated the best final biogas composition.

The AHPD concept has also shown another advantage—the biogas produced in both reactors has very different compositions. It opens up new possibilities; for example, using the high-methane product from the first reactor for energy generation, while the biogas from the second tank can be used to process heating. These small improvements in flux management could potentially increase the economical profitability of the plant.

Regarding the TPAD process, the production was more efficient than in the classical biogas plant. The biggest increase can be noticed for manure-diluted feedstock reactors, reaching ~9.6%. However, this improvement has its price, as the methane content was distinctly lower. In the most utter case, the drop was around 8.66%, which makes a huge difference in the economic feasibility of this concept. Therefore, further biogas upgrading can be expensive. The summary of both the volumetric and compositional efficiency is summarized in Table 3.

**Table 3:** Final comparison of methods

		Biogas [Nm$^\mathbf{3 }$/d]	Methane [%]		Biogas [Nm$^\mathbf{3 }$/d]	Methane [%]
Case 1 (51.61% MS, 48.39% W)				Case 2 (52.38% MS, 47.62% W)		
Initial		11,916.36	55.36		12,052.70	55.34
Change	AHPD TPAD	-6.58 [%] 9.08 [%]	7.35 [%]-8.28 [%]	AHPDTPAD	-6.14 [%]9.06 [%]	7.30 [%]-8.27 [%]
Case 3 (50.82% MS, 49.18% PM)				Case 4 (51.61% MS, 48.39% PM)		
Initial		11,786.09	56.42		11,908.66	56.36
Change	AHPD TPAD	-7.97 [%]9.59 [%]	8.50 [%]-8.66 [%]	AHPDTPAD	-7.59 [%]9.55 [%]	8.42 [%]-8.63 [%]

The presented approach was intended to test if the proposed modification can be beneficial on a full industrial scale. As it was already mentioned, the presented configurations are still uncommon in systems larger than the pilot-size [21, 22]. The developed tool proved to be able to build recommendations basing on available data, which can be a good start point in the design of the real installation or further experiments. However, it has to be mention, that the method indicated in the study has several limitations. Firstly, the model generates reliable results only under certain conditions. There is no compelling evidence if it can be applicable for a system that varies significantly from the presented one; e.g., with untypical feedstock, with other modifications in the production step, under untypical temperatures, etc. The second aspect is the limitations of precision. As Table 1 shows, the relative error does not exceed 5%, which is a satisfactory result for preliminary screening. Despite this, the final application could also be preceded by at least a pilot-scale test.

## Conclusions

The modified and verified mathematical model of the AD process proved to be a valuable tool for the description and design of biogas plant technological installations, at least under the conditions in which it was tested. Its analysis allowed for the prediction of process efficiency results and provided many suggestions and specific installation design solutions that lead to process optimization.

The analysis shows that the overall impact of the presented process modifications is mutually opposite. While the overpressure leads to a significant improvement in biogas composition, it also negatively affects the production efficiency. On the other hand, the TPAD system indicated the best volumetric flux of produced fuel; however, at the same time, it was characterized by the lowest methane content within the considered configurations.

The feedstock composition has a moderate and unsteady impact on the production profile, in the tested modifications. The dilution with pig manure, instead of water, leads to a slightly better efficiency in the classical configuration, while the impact on the biogas composition was negligible. Meanwhile, for the TPAD process, the trend is very similar, but the AHPD biogas plant indicates a reverse tendency. Besides, the manure-diluted feedstock, in this configuration, resulted in a noticeably better biogas composition.

Overall, the recommendation from this article is to use the AHPD concept if the composition of the biogas is the most important factor—when gathering the fuel only from the first tank, a methane content value even higher than 82% can be achieved. In the case in which the performance is the most important factor, it is favorable to use the TPAD configuration. It could potentially be beneficial to combine these two processes (temperature and pressure phasing) to take the best from both approaches. However, at this stage, the model is not designed to simulate this type of process; thus, it was not considered in this work.

## Methods

### Initial data structure and sources

**Fig. 2:**
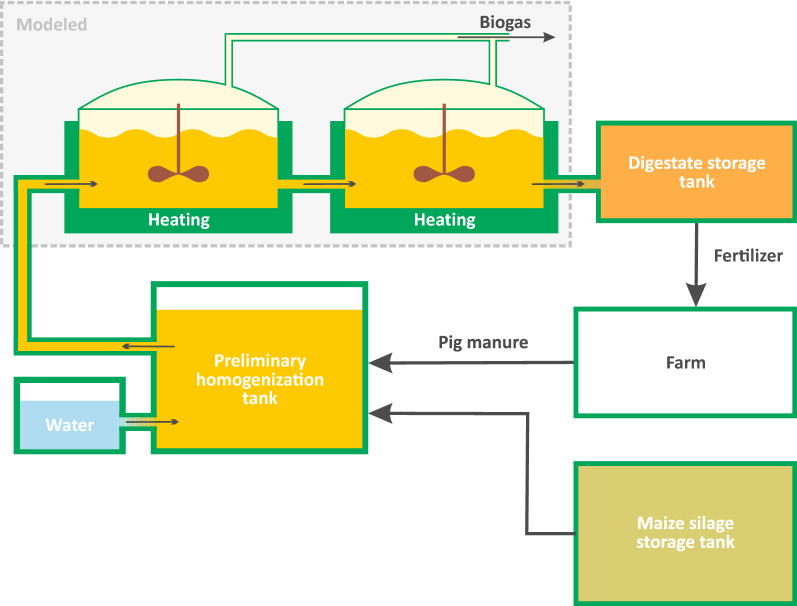
Configuration of the tested biogas plant

As a reference, literature data about rural biogas plant located in Poland was selected [29]. This unit works with a mixed substrate consisting of maize silage, diluted by water and pig manure, in varying proportions. The production chain contains two reactors working in continuous mode, in series, each having a total volume of 3,300 $\hbox {m}^{3}$. The biogas from each tank was collected separately, to membrane reservoirs, located under the tanks. The hydraulic retention time (HRT) of the substrate in every chamber was 32 days, while the temperature was kept constant, namely at 39$^{\circ }$C. No additional modifications, such as pH control or overpressure, were made. The scheme of the process is presented in Fig. 2. Overall, the experiment lasted one month, during which four feedstock mixtures were tested.

The composition of the feedstock changed sequentially. The available data included 4 possible feeding options, containing: maize silage, pig manure, and water. The four feeding options had the following compositions of these materials: (1) 51.61%, 0%, 48.39%; (2) 52.38%, 0%, 47.62%; (3) 50.82, 49.18%, 0%; and (4) 51.61%, 48.39, 0%. The HRT was kept constant, independently from the composition of the feedstock. The mixture was homogenized before being pumping into the reactor to avoid any composition fluctuations.

The properties of the feedstock were calculated based on a statistical analysis, following the methodology described in our previous work [27]. Briefly, the results from extended Weende analysis of selected types of raw materials [30–33] were used to calculate the overall feeding value of biomass (*X*$_{\mathrm {c}}$) and of the four fractioning factors (proteins, lipids, carbohydrates, and inert fraction). The summary of the basic configuration is presented in Table 4.

**Table 4:** Initial model configuration

	Description	Case 1	Case 2	Case 3	Case 4
Feedstock composition [%]		51.61 MS + 48.39 W	52.38 MS +47.62 W	50.82 MS +49.18 PM	51.61 MS +48.39 PM
${X}_{{c}}$ [kgCOD/$\hbox {m}^{3}$]	Feeding value	283.48	286.61	331.95	334.10
${f}_{{pr}}$ [-]	Protein	0.1105	0.1105	0.1272	0.1268
${f}_{{li}}$ [-]	Lipid	0.0547	0.0547	0.0495	0.0496
${f}_{{ch}}$ [-]	Carbohydrate	0.6998	0.6998	0.5721	0.5746
${f}_{{ine}}$ [-]	Inert	0.1360	0.1360	0.2519	0.2497
${T}_{{1}}$ [$^{\circ }$C]	Temperature in R1	39			
${T}_{{2}}$ [$^{\circ }$C]	Temperature in R2	39			
${V}_{{R1}}$ [$\hbox {m}^{{3}}$]	Volume of R1	3,300			
${V}_{{R2}}$ [$\hbox {m}^{{3}}$]	Volume of R2	3,300			
${F}_{{IN}}$ [$\hbox {m}^{{3}}$/d]	Input feedstock flux	87.5			

*MS* maize silage, *W* water, *PM* pig manure

### Model characteristics and implementation of process modifications

A typical biogas plant is expected to perform all stages of biochemical conversion—from hydrolysis to methanogenesis—in a single reactor. In a more complex installation, like the one considered in this study, two reactors operating under the same conditions can be considered. This system requires the use of average values of process parameters, selected so that all stages of the conversion can take place with satisfactory performance. However, it seems that the process efficiency can be significantly improved by including two reactors in series in the biogas plant design.

This makes it possible to vary the process conditions (temperature, pressure, and pH) for the successive stages. The conditions in the first reactors could favor hydrolysis and, to a lower extent, acido- and acetogenesis. In the second stage, the environment should facilitate the growth of archaeons, which will carry out methanogenesis.

The design and analyses of this study were based on an AD model developed earlier [28, 34], that was partially based in its assumptions on the universal ADM1 tool [35]. The implementation of the ADM1 for the design and control of innovative reactor types lies within its field of application, although it is still not widespread. This is due to the complexity of the system, the lack of an appropriate database of proper substrate characteristics, and the sensitivity of the model parameters to specific substrates, or to operational conditions changes. Our model is a mathematical description of the most important unit processes occurring during AD of organic compounds to methane, with the participation of microorganisms (Fig. 1). The modified system of biochemical transformations includes 26 components in the liquid phase: dissolved (14), undissolved (5), and 7 groups of microorganisms, as well as 3 gas components. The model includes the processes of association and dissociation with hydrogen and hydroxide ions in the liquid phase, which allowed the determination of the pH. Methane, hydrogen, and $\hbox {CO}_{{2}}$ present in the gas phase are the final products of the process and their measured amounts depend on the process efficiency.

General mass balance equation for a continuous reactor with ideal (continuous) mixing Eq. (1):

1 $$\begin{aligned}{[Accumulation \; of \; mass]} = {[input]}\; - \;{[output]} \;+\; {[production]} \end{aligned}$$

After applying this equation to each of the components present in the liquid phase of the system, it takes the form of Eq. (2).

2 $$\begin{aligned} \frac{{\text{d}}C_{x}}{{\text{d}}t}=\frac{F_{IN}\cdot C_{x\_ in}-F_{OUT}\cdot C_{x}}{V_{w}}+\sum _{j=1}^{n}v_{i,j}\cdot r_{j}, \end{aligned}$$

where $\mathbf{{C}_{{x}}{, C}_{{x\_in}}}$ define the concentration of a component in the liquid phase inside the reactor, and the input concentration of a component, respectively. $\mathbf{{V}_{{w}}}$ refers to the working volume of the reactor, and $\mathbf{{F}_{{IN}}}$ = $\mathbf{{F}_{{OUT}}}$ describes the volumetric flux to and from the reactor. The last part of the equation sums the reaction rates of all unit processes (r$_{{j}}$), multiplied by the stoichiometric factors ${(v}_{{i,j}})$.

In the first reactor of the two-stage process (Fig. 2), for all components that are included in the model, except for raw biomass, is ${C}_{{x\_in}}$ is equal to 0, because at the entrance of this reactor, the raw biomass has not yet been hydrolyzed.

In the second tank, the change in the concentration of any component is determined using Eq. (3):

3 $$\begin{aligned} \frac{{\text{d}}C_{x}}{{\text{d}}t}=\frac{F_{OU{T_{R1}}}\cdot C_{{x_{OU{T_{R1}}}}}-F_{OUT}\cdot C_{x}}{V_{w}}+\sum _{j=1}^{n}v_{i,j}\cdot r_{j} \end{aligned}$$

In contrast to the corresponding relationship for the first tank (Eq. (2)), the inflow of each component will be different from zero ($F_{OU{T_{R1}}}$) and it depends on the concentration after the first stage ($C_{{x_{OU{T_{R1}}}}}$) The concentration values of all composites (C$_{ {x\_in}}$) at the inlet of the second tank are equal to the respective concentrations at the outlet of the first reactor ($C_{{x_{OU{T_{R1}}}}}$). However, since the reactions take place in the liquid phase in both reactors: ${F}_{\mathrm {in}}$ = ${F}_{\mathrm {out}}$. The second part of Eq. (2) and Eq. (3) describes the conversions that generate or consume a selected component of the system, including biotransformation, a physical process, such as the transfer of a component on the border of phases, as well as the decay of microorganisms.

The mass balance of the gaseous components ($\hbox {CH}_{{4}}$, $\hbox {CO}_{{2}}$, and $\hbox {H}_{{2}}$), which are products that result from fermentation, is reduced to similar ordinary differential equations (ODEs, Eq. (2)), assuming that ${F}_{\mathrm {in}}$ = 0, and by replacing the reactor working volume in equation (${V}_{\mathrm {w}}$) by the volume of the gas phase in the reactor (${V}_{\mathrm {g}}$). The last part of Eq. (2), which refers to the biochemical conversions, will be replaced by the kinetic rate of the gas transfer between the liquid and the gas phases (the intensity of gas transfer, $lgt_{{CH_{4}}}$). For methane (${CH}_{{4}}$), the equation will take the following form:

4 $$\begin{aligned} \frac{{\text{d}}S_{{CH_{4}}\_ g}}{dt}=\frac{0-S_{{CH_{4}}\_ g}\cdot F_{OUT\_ g}}{V_{g}}+lgt_{{CH_{4}}}\cdot \frac{V_{w}}{V_{g}}, \end{aligned}$$

where $\mathbf{{S}_{{CH_4}\_g}}$ is the methane concentration [$mol/{m}^{3}$], $\mathbf{{V} _{w } /V _{g }}$ is the proportion between the liquid and the gas phase, $\mathbf{{F} _{OUT\_g }}$ describes the volumetric flux of the collected biogas, and $\mathbf{{ lgt }_{{{ CH }_{4 }}}}$ is the kinetic rate of $\hbox {CH}_{{4}}$ transfer (“the speed of gas transfer”) between the liquid and the gas phases in the anaerobic reactor. The detailed equation for this parameter is presented below:

5 $$\begin{aligned} F_{OUT\_ gas}=k_{p}\cdot V_{g}\cdot (P_{Tot}-P_{atm})\cdot P_{Tot}/P_{atm}, \end{aligned}$$

where $\mathbf{{k} _{p }}$ refers to a parameter related to the outlet pipe resistance (in this study, it assumed as $1 {\times } 10^{-4} [1/(\hbox {d}{\cdot }\hbox {bar})])$, and $\mathbf{{P} _{Tot }}$ is the sum of the partial pressures of all gases in the digester.

While the above-described model is correct for an atmospheric pressure and a constant temperature process, it needed to be adjusted to be valid for novel AD modifications [36, 37], which we intended to examine in this article. Equation (5) needs to be modified by replacing the $\hbox {P}_{\mathrm {atm}}$ (atmospheric pressure) by $\hbox {P}_{\mathrm {OP}}$ (overpressure), which will be achieved by the delay in the discharge of the biogas from the reactor chamber:

6 $$\begin{aligned} F_{OUT\_ gas}=k_{p}\cdot V_{g}\cdot \left( P_{Tot}-P_{OP}\right) \cdot P_{Tot}/P_{OP}. \end{aligned}$$

Such simulation will allow to check the concept of the AD process carried out under overpressure—AHPD. To properly assess the impact of pressure on the process and compare its efficiency with that of a classical process, a conversion of the output biogas flux to normal conditions was introduced in the model (Eq. (7)). The gas from both tanks is received independently; thus, the ODEs for gaseous substances do not change and are identical for both reactors.

7 $$\begin{aligned} F_{OUT\_ gas\_ N}=\frac{P_{OP}\cdot F_{OU{T_{gas}}}\cdot 273.15\,K}{T\cdot 1.01325\cdot 10^{5}Pa}. \end{aligned}$$

It would be expected that, by carrying out the process in two steps, and working with an elevated pressure in the first tank and with atmospheric pressure in the second, would allow an optimal use of the biomass and improve the quality of the obtained biogas.

The TPAD model includes thermal phasing. The transformations in the first reactor are carried out at higher temperatures (55 - $65^{\circ }\hbox {C}$), which favor thermophilic bacteria and accelerate biomass hydrolysis. In the second reactor, a temperature suitable for mesophilic bacteria ($35^{\circ }\hbox {C}$) is kept. The set of model equations for both stages (reactors) is the same, while there are differences in the process constants, which are dependent on temperature. Assuming the exponential dependence of these constants with temperature and making them depend on the initial known constant value, one can write equation:

8 $$\begin{aligned} k_{x\_ T}=k_{x\_ T0}*e^{\theta \left( T-{T_{0}}\right) }, \end{aligned}$$

where $\mathbf{{k} _{x\_T0 }}$ is the value of the selected constant, for a temperature of $\hbox {T}_{{0}}$ = 35$^{\circ }$C. The T means the exact temperature of the process. The $\uptheta$ refers to the coefficient of temperature-dependency for the final **k**$_\mathbf{x\_T }$ value.

By knowing the values of the reaction rate constants at two different temperatures after transforming Eq. (8), it is possible to determine the value of the temperature coefficient:

9 $$\begin{aligned} \theta =\frac{\ln \frac{k_{x\_ T}}{k_{x\_ T0}}}{\left( T-T_{0}\right) }. \end{aligned}$$

The initial (basic model), the adjusted values of the selected constant, and its ${\uptheta }$ are presented in Table 5. Three parameters were considered: propionate ($\mathbf{k} _\mathbf{m\_pro }$) and acetate ($\mathbf{k} _\mathbf{m\_ac }$) conversion rates, and the hydrolysis rate ($\mathbf{k} _\mathbf{dis }$). These rates are calculated independently for both tanks, as both have different reaction temperatures. From the value of the coefficient, a few conclusions can be withdrawn: first of all, the hydrolysis will be more efficient at a higher temperature, which complies with the behavior of a real TPAD plant. The methanogenesis will be favored in the second reactor, where the temperature is lower, as the ${\uptheta }$ values for $\mathbf{k} _\mathbf{m\_pro}\; \text{and}\; \mathbf{k} _\mathbf{m\_ac }$ are positive.

**Table 5:** Summary of the parameters optimized in the TPAD model

Reaction rate	Initial value	Optimized value for $\hbox {T}_{{0}}$ (35$^{\circ }$C)	Temperature dependency coefficient
	[**d**$^\mathbf{-1 }$]	[**d**$^\mathbf{-1 }$]	$\varvec{\Theta }$ [$^{\circ }\hbox {C}^\mathbf{-1 }$]
*k*$_\mathbf{m\_pro }$	13.0	7.19	0.02615
k$_\mathbf{m\_ac }$	8.0	14.27	0.00434
k$_\mathbf{dis }$	0.5	1.105	-0.06879

### The procedure of testing alternative configurations

**Fig. 3:**
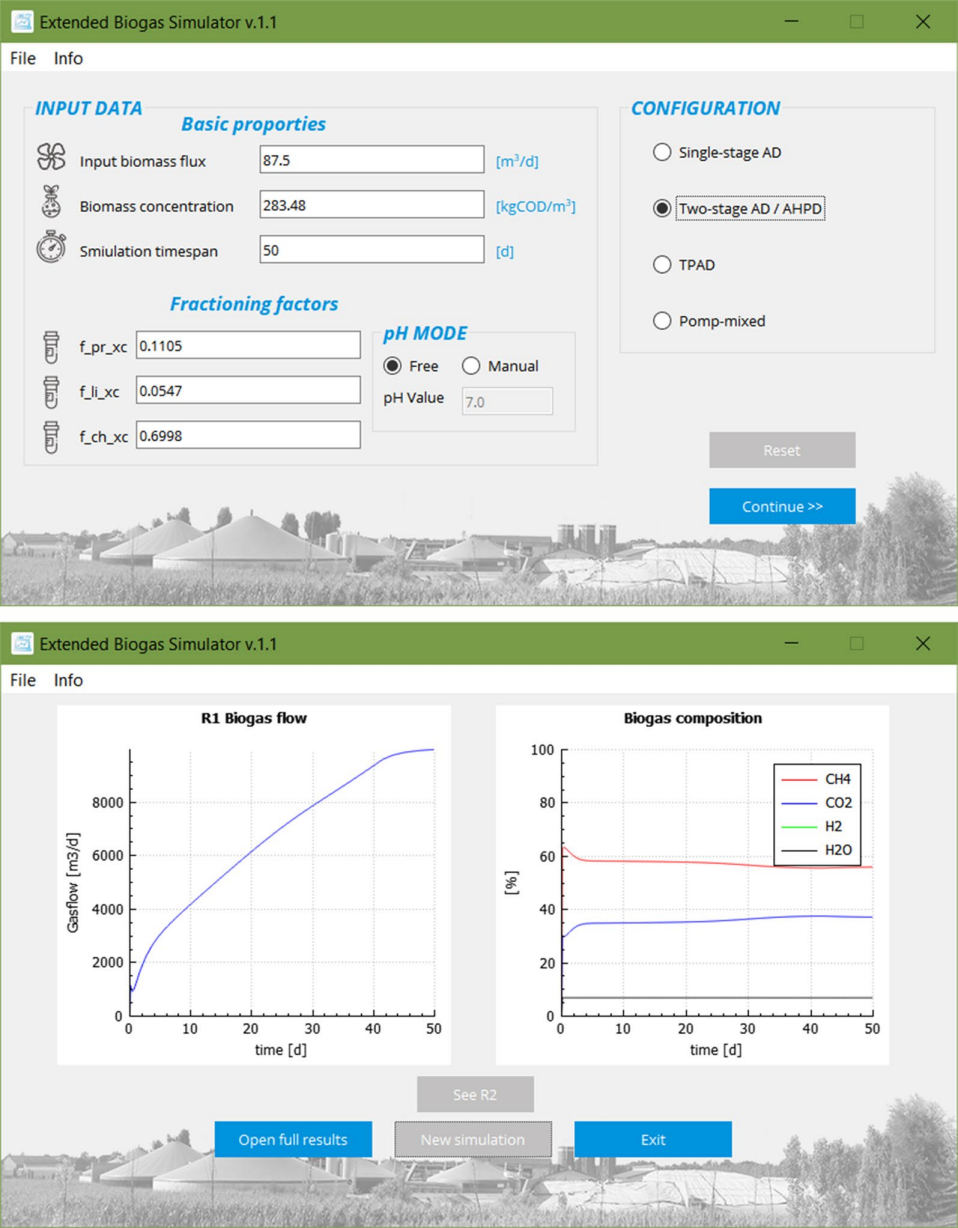
GUI of the final model

In section 2.1, the configuration for model validation (Table 4) was presented. However, the aim of this article is to analyze if alternative concepts can achieve a better performance or biogas composition. As it was already mentioned, two types of novel solutions were selected—AHPD and TPAD —and implemented in the model (Fig. 3). To keep the result the most reliable, the 4 feedstock compositions, and input fluxes are kept the same. The pressure, temperature, or pH differed, depending on the tested configuration.

In modification 1, the AHPD process is examined. The pressure in the first tank is assumed to be 20 times higher (20.27 bar) than that in the reference test, while in the second tank, it is kept unchanged (1.013 bar). As the overpressure leads to a higher solubility of carbon dioxide, carbonic acid will be formed, leading to a significant drop in the pH value. To resolve this issue, the pH needs to be controlled. The value of this parameter is selected based on the initial tests (Table 4). The steady-state pH values of every initial case (C1 to C4) were introduced in this test. These were: 7.37, 7.36, 7.45, and 7.44, respectively.

In modification 2 (TPAD), the pressure remained the same as in the initial test. The temperature was raised to 55$^{\circ }$C in the first reactor, while in the second, it was kept at the initial value: 39$^{\circ }$C. As there was no overpressure, the pH control was omitted. The acidity of the environment changed freely, as in the reference test.

## Data Availability

All data generated or analyzed during this study are included in this published article.

## Abbreviations

$\Theta$: Temperature coefficient for $\hbox {k}_{\mathrm {x}}$
AD: Anaerobic digestion
AHPD: Autogenerative high-pressure digestion
AT-TPAD: TPAD with acid pH in the first stage
C$_{\mathrm {X}}$: The concentration of substance X
F$_{\mathrm {IN}}$: Input flux to the reactor
F$_{\mathrm {OUT}}$: Output flux from the reactor
f$_{\mathrm {x}}$: Fractioning factor to group x
k$_{\mathrm {p}}$: Outlet pipe resistance parameter
k$_{\mathrm {x\_Tn}}$: Reaction rate at temperature n
lgt: Liquid–gas transfer rate
NT-TPAD: TPAD with neutral pH in the first stage
P: Pressure
R1, R2: Reactor 1 or 2
S$_{\mathrm {x}}$: Soluble or gas substance concentration
T: Temperature
TPAD: Two-phase anaerobic digestion
V$_{\mathrm {g}}$: Gas phase volume
V$_{\mathrm {R}}$: Total volume of the reactor
V$_{\mathrm {w}}$: Working volume of the reactor
X$_{\mathrm {c}}$: Total feeding value of biomass
